# Initial value problems for system of differential-algebraic equations in Maple

**DOI:** 10.1186/s13104-018-3748-0

**Published:** 2018-09-06

**Authors:** Srinivasarao Thota

**Affiliations:** Department of Applied Mathematics,School of Applied Natural Sciences, Adama Science and Technology University, Post Box No. 1888, Adama, Ethiopia

**Keywords:** Initial value problems, Differential-algebraic systems, Symbolic algorithm, Green’s function, Maple packages, 34A09, 65L05, 15A29

## Abstract

**Objectives:**

In this paper, we discuss a Maple package, deaSolve, of the symbolic algorithm for solving an initial value problem for the system of linear differential-algebraic equations with constant coefficients.

**Results:**

Using the proposed Maple package, one can compute the desired Green’s function of a given IVP. Sample computations are presented to illustrate the Maple package.

## Introduction

Applications of DAEs arise naturally in many fields, for example, various dynamic processes, mechanical systems, simulation of electric circuits and chemical reactions subject to invariants etc., and these are often expressed by DAEs, which consist of algebraic equations and differential operations. Several methods and algorithms have been introduced by many researchers and engineers to solve the IVPs for systems of DAEs. Most of them are tried to find an approximate solution of the given system. However, we recall a symbolic algorithm to compute the exact solution of a given system of DAEs (See [1] for further details of the algorithm). In this paper, we discuss the Maple package of the symbolic algorithm that computes the exact solution.

There are several implemented methods available in various mathematical softwares tools like Matlab, Mathematica, SCIlab etc. All these implementations are applied to find the general solution of a given system DAEs with free parameters. Then, we could find values of parameters by substituting the initial conditions. For example, the implemented method in Mathematica is based on decomposing the coefficient matrices, *A* and *B*, into nonsingular and nilpotent part. Then generalized inverse for *A* and *B* is calculated, and the problem is reduced to solving a system of ODEs. So existing solvers for ODEs can be used. In Matlab, the equation is also converted to system of ODEs by reducing the differential index and then we find the general solution with free parameters. However, in the proposed algorithm, we compute the exact solution directly without free parameters. The implemented Maple packege is based on the convertion of the given system into a canonical form using the *shuffle algorithm* which produces another simple equivalent system, and the canonical system can be solved easily. The Maple implementation includes computing the canonical system and the exact solution of a given IVP. The comparison of the implemented Maple package with existing methods implemented in other mathematical software like Matlab and Mathematica is also discussed in Results Section. In this paper, we focused on Maple implementation of an IVP with homogeneous initial conditions, however we also discuss an algorithm to check the consistency of the non-homogeneous initial conditions.

### Symbolic algorithm of IVPs for systems of lDAEs

In this paper, we focused on a system of DAEs has the general form

1 $$\begin{aligned} A{\texttt{D}}y(x)+By(x)=f(x). \end{aligned}$$

The system (1) is purely algebraic system if $A = 0$, and there exist many methods and algorithms to compute all possible solutions, see, for example, [2, 3]. The system (1) becomes a system of ordinary differential equations if $\det (A) \ne 0$ (we call *A* is regular matrix), and the solution of first order system of LDEs is discussed in [2–9]. Therefore, we focused on a system of the form (1) where the coefficient matrix *A* is non-zero singular matrix. Suppose $\mathcal {F} = C^\infty [a,b]$ for simplicity, and $[a,b] \subset \mathbb {R}$. Now the operator form of an IVP for DAEs can be represented as

$$\begin{aligned}&Ly = f, \\&{\texttt{E}}y = 0, \end{aligned}$$

where $L = A{\texttt{D}}+ B \in \mathcal {F}^{n \times n}[{\texttt{D}}]$ is the matrix differential operator, ${\texttt{D}}= \frac{d}{dx}$, $y \in \mathcal {F}^n$ is unknown vector to be determined, $f \in \mathcal {F}^n$ is the vector forcing function and $\texttt{E}$ is the evaluation operator. We want to find the matrix Green’s operator $G \in \mathcal {F}^{n \times n}[{\texttt{D}},{\texttt{A}}]$ such that $Gf = y$ and ${\texttt{E}}G = 0$.

The following Lemma 1 is one of the essential steps for the proposed algorithm. The lemma gives the variation of parameters formula of an IVP for higher-order scalar linear differential equations over integro-differential algebras.

#### **Lemma 1**

(Thota and Kumar   [1, 2, 5, 7, 9])   Let $(\mathcal {F}, {\texttt{D}}, {\texttt{A}})$* be an ordinary integro-differential algebra. Suppose* $T = {\texttt{D}}^m + a_{m-1}{\texttt{D}}^{m-1} + \cdots + a_0 \in \mathcal {F}[{\texttt{D}}]$ *is a monic scalar differential operator of order m and* $v_1, \ldots , v_m$ *is fundamental system for T. Then the right inverse operator of T is given by*

2 $$\begin{aligned} T^\divideontimes = \sum \limits _{i=1}^{m} v_i {\texttt{A}}w^{-1}w_i \in \mathcal {F}[{\texttt{D}},{\texttt{A}}], \end{aligned}$$

*where w is the determinant of the Wronskian matrix W for* $v_1, \ldots , v_{m}$ and $w_i$* the determinant of the matrix* $W_i$* obtained from W by replacing the i*-*th column by m-th unit vector.*

In order to obtain the Green’s function and the exact solution of a given system of DAEs, we first find a canonical form of the given DAEs system using the shuffle algorithm [1, 10] that transforms the given system into another equivalent and simpler system that can be solved easily. The Green’s operator and Green’s function of the given system of DAEs with initial conditions, with the help of the canonical form, are computed in the following theorem.

#### **Theorem 2**

(Thota and Kumar [1]) * Let* ($\mathcal {F}, {\texttt{D}}, {\texttt{A}}$)* be an ordinary integro-differential algebra. Let* $\tilde{L} = \tilde{A}{\texttt{D}}+ \tilde{B} \in \mathcal {F}^{n \times n}[{\texttt{D}}]$* be the canonical form of* $L = A{\texttt{D}}+B$* with initial conditions; and* $\{v_1, \ldots , v_{n}\}$* be a fundamental system for* $T = \det (\tilde{L})$.* Then the regular IVP for system of DAEs*

$$\begin{aligned}&Ly = f, \\&{\texttt{E}}y = 0, \end{aligned}$$

*has the unique solution*

3 $$\begin{aligned} y = \begin{pmatrix} \sum _{i=1}^{n} (-1)^{i+1} \mathcal {L}_i^1 T^\divideontimes \tilde{f_i} \\ \vdots \\ \sum _{i=1}^{n} (-1)^{i+n} \mathcal {L}_i^n T^\divideontimes \tilde{f_i} \end{pmatrix}, \end{aligned}$$

*where* $\mathcal {L}_i^j$* is the determinant of* $\tilde{L}$ *after removing i-th row and j-th column;* $T^\divideontimes$ *is the right inverse of T; and* $\tilde{f} = (\tilde{f_1}, \ldots , \tilde{f_n})^t$.* The Green’s operator is*

4 $$\begin{aligned} G = \begin{pmatrix} (-1)^{1+1} \mathcal {L}_1^1 T^\divideontimes &{} \cdots &{} (-1)^{n+1} \mathcal {L}_n^1 T^\divideontimes \\ \vdots &{} \ddots &{} \vdots \\ (-1)^{1+n} \mathcal {L}_1^n T^\divideontimes &{} \cdots &{} (-1)^{n+n} \mathcal {L}_n^n T^\divideontimes \end{pmatrix} \end{aligned}$$

*such that* $G\tilde{f} = y$* and* ${\texttt{E}}~G = 0$.

### Non-homogeneous initial conditions

In general, there is no freedom to choose non-homogeneous initial conditions for proposed method. Hence, in [1, Proposition 2.5] authors presented an algorithm to check the consistency of a given non-homogeneous initial conditions. In this section, we recall the algorithm in Proposition 3 to check the consistency of the non-homogeneous initial conditions.

#### **Proposition 3**

(Thota and Kumar [1]) * Let* ($\mathcal {F}, {\texttt{D}}, {\texttt{A}}$)* be an ordinary integro-differential algebra. Suppose* $\tilde{T} = \tilde{A}{\texttt{D}}+ \tilde{B} \in \mathcal {F}^{n \times n}[{\texttt{D}}]$* is a canonical form of* $T = A{\texttt{D}}+B$* with non-homogeneous initial conditions. The non-homogeneous initial condition* ${\texttt{E}}u = \alpha$* is consistent, if*

5 $$\begin{aligned} UU_a^{-1}\alpha \in \text {Ker}(T), \end{aligned}$$

*where U is the fundamental matrix of* $\tilde{T}$* and* $U_a$ *is the value of U at initial point a*.

The following example shows the computation of the exact solution using the algorithm presented in Theorem 2, and also check the consistency of the non-homogeneous initial conditions using the algorithm presented in Proposition 3.

#### *Example 4*

Consider the following differential-algebraic equations.

6 $$\begin{aligned}y_1'+y_2'+y_1+y_2&=x \\y_1-y_2 &= \sin x \end{aligned}$$

with initial condition $y_1(0)=y_2(0)=0$.

The matrix differential operator and the canonical form of  (6) are

$$\begin{aligned}T = \begin{pmatrix} 1+{\texttt{D}}~&{}~ 1+{\texttt{D}}\\ 1 &{} -1 \end{pmatrix},~~~ \tilde{T} = \begin{pmatrix} {\texttt{D}}&{} 2+{\texttt{D}}\\ {\texttt{D}}&{} -{\texttt{D}}\end{pmatrix} ~~\text {and}~~ \tilde{f} = \begin{pmatrix} x-\sin x \\ \cos x \end{pmatrix}. \end{aligned}$$

Following the algorithm in Theorem 2, we have the exact solution

7 $$\begin{aligned} y = \begin{pmatrix} \frac{1}{2}e^{-x} + \frac{1}{2} \sin x + \frac{1}{2} x - \frac{1}{2}\\ \frac{1}{2}e^{-x} - \frac{1}{2} \sin x + \frac{1}{2} x - \frac{1}{2} \end{pmatrix}. \end{aligned}$$

One can easily check that $Ty = f$ and ${\texttt{E}}y = 0$.

Consider the non-homogeneous initial conditions $y_1(0) = \alpha , y_2(0)=\beta$ with given system (6). From Proposition 3, the initial conditions are consistent if $UU_0^{-1} \alpha \in \text {Ker}(T)$, where

$$\begin{aligned} U=\begin{pmatrix} 1 &{} e^{-x} \\ 0 &{} e^{-x} \end{pmatrix}, U_0= \begin{pmatrix}1 &{} 1 \\ 0 &{} 1 \end{pmatrix},~~\text {and}~~ \alpha = \begin{pmatrix}\alpha \\ \beta \end{pmatrix}. \end{aligned}$$

Now

$$\begin{aligned} UU_0^{-1} \alpha = \begin{pmatrix} 1 &{} e^{-x} \\ 0 &{} e^{-x} \end{pmatrix} \begin{pmatrix}1 &{} -1 \\ 0 &{} 1 \end{pmatrix} \begin{pmatrix}\alpha \\ \beta \end{pmatrix} = \begin{pmatrix} e^{-x}\beta + \alpha - \beta \\ e^{-x}\beta \end{pmatrix} \end{aligned}$$

and

$$\begin{aligned} T(UU_0^{-1} \alpha ) = \begin{pmatrix} 1+{\texttt{D}}~&{}~ 1+{\texttt{D}}\\ 1 &{} -1 \end{pmatrix} \begin{pmatrix} e^{-x}\beta + \alpha - \beta \\ e^{-x}\beta \end{pmatrix} = \begin{pmatrix} \alpha -\beta \\ \alpha -\beta \end{pmatrix}, \end{aligned}$$

which gives $\alpha - \beta = 0$ for $UU_0^{-1} \alpha \in \text {Ker}(T)$, and hence the consistent initial conditions are $y_1(0) = \alpha ,y_2(0)=\beta$, such that $\alpha -\beta =0$. The solution $y_c$ of the IVP $Ty=0, {\texttt{E}}y = (\alpha ,\beta )^T$ computed as (see [1] for more details),

$$\begin{aligned} y_c = UU_0^{-1} \alpha = \begin{pmatrix} e^{-x}\beta + \alpha - \beta \\ e^{-x}\beta \end{pmatrix}, \end{aligned}$$

and from (7)

$$\begin{aligned}y_p=\begin{pmatrix} \frac{1}{2}e^{-x} + \frac{1}{2} \sin x + \frac{1}{2} x - \frac{1}{2}\\ \frac{1}{2}e^{-x} - \frac{1}{2} \sin x + \frac{1}{2} x - \frac{1}{2} \end{pmatrix}. \end{aligned}$$

The exact solution of the regular system

$$\begin{aligned} \left( {\begin{array}{*{20}c} {1 + {\texttt{D}}} & {1 + {\texttt{D}}} \\ 1 & { - 1} \\ \end{array} } \right)\left( {\begin{array}{*{20}c} {y_{1} } \\ {y_{2} } \\ \end{array} } \right) & = \left( {\begin{array}{*{20}c} x \\ {\sin x} \\ \end{array} } \right), \\ E\left( {\begin{array}{*{20}c} {y_{1} } \\ {y_{2} } \\ \end{array} } \right) & = \left( {\begin{array}{*{20}c} \alpha \\ \beta \\ \end{array} } \right),\,\,\alpha - \beta = 0, \\ \end{aligned}$$

is $$y=y_c+y_p$$, i.e.,

$$\begin{aligned} y = \begin{pmatrix} \frac{1}{2}e^{-x} + \frac{1}{2} \sin x + \frac{1}{2} x - \frac{1}{2}+e^{-x}\beta + \alpha - \beta \\ \frac{1}{2}e^{-x} - \frac{1}{2} \sin x + \frac{1}{2} x - \frac{1}{2}+e^{-x}\beta \end{pmatrix}. \end{aligned}$$

## Main text

In this section, we discuss the Maple package daeSolve to solve the IVPs for system of DAEs.

### Algorithm

#### Canonical matrix differential system

The following algorithm produces the canonical form of a given system of DAEs.

**Input:** Coefficient Matrices *A*, *B* and vector function *f*.
**Output:** Canonical system $\tilde{L}$ and $\tilde{f}$.

##### **Algorithm 1**

1. $$M \leftarrow \left(\begin{array}{*{20}c} A & \vdots & B & \vdots & f \\ \end{array}\right )\,(Augmented\,matrix)$$.
2. *r* ← 0
3. **Do**
4. *M_reduced* ← *ReducedRowEchelonForm*(*M*)
5. $$A\_reduced \leftarrow \left( {\begin{array}{*{20}c} {A{}_{1}} \\ 0 \\ \end{array} } \right),B\_reduced \leftarrow \left( {\begin{array}{*{20}c} {B{}_{1}} \\ {B{}_{2}} \\ \end{array} } \right),f\_reduced \leftarrow \left( {\begin{array}{*{20}c} {\widehat{{f{}_{1}}}} \\ {\widehat{{f{}_{2}}}} \\ \end{array} } \right)$$
6. $$\tilde{A} \leftarrow \left( {\begin{array}{*{20}c} {A_{1} } \\ {B_{2} } \\ \end{array} } \right),\tilde{B} \leftarrow \left( {\begin{array}{*{20}c} {B_{1} } \\ 0 \\ \end{array} } \right),\tilde{f} = \left( {\begin{array}{*{20}c} {\widehat{{f_{1} }}} \\ {\frac{d}{dx}\widehat{{f_{2} }}} \\ \end{array} } \right)$$
7. *M *← *M_reduced*
8. $$r \leftarrow rank(\tilde{A})$$
9. **While** (*r *< *n*)
10. $$\tilde{L} \leftarrow \tilde{A} \cdot {\text{D}} + \tilde{B},\tilde{f}$$

#### Exact solution

The following algorithm produces the exact solution of a given system of DAEs.

**Input:** Coefficient Matrices *A*, *B* and vector function *f*.
**Output:** Exact Solution

##### **Algorithm 2**

1. *L*, *F *← *CanonicalMatrixDiffSys*(*A*,*B*,*f*).
2. *Adj_L* ← *Adjoint Matrix of L*
3. *Det_L* ← *Determinant of L*
4. *Det_Sol* ← *Solve linear DE* (*dsolve*(*Det_L*, *ics*))
5. *Exact_Sol* ← *Adj_L *∙ *Det*_*Sol *∙ *F*

### Maple package, deaSolve, for IVPs for system of DAEs

In this section, we present the Maple procedures of the proposed algorithm. The data type MatrixDiffOperator(A,B) is created to generate the matrix differential operator *L* of a given system, where *A* and *B* are the coefficient matrices of a given system.
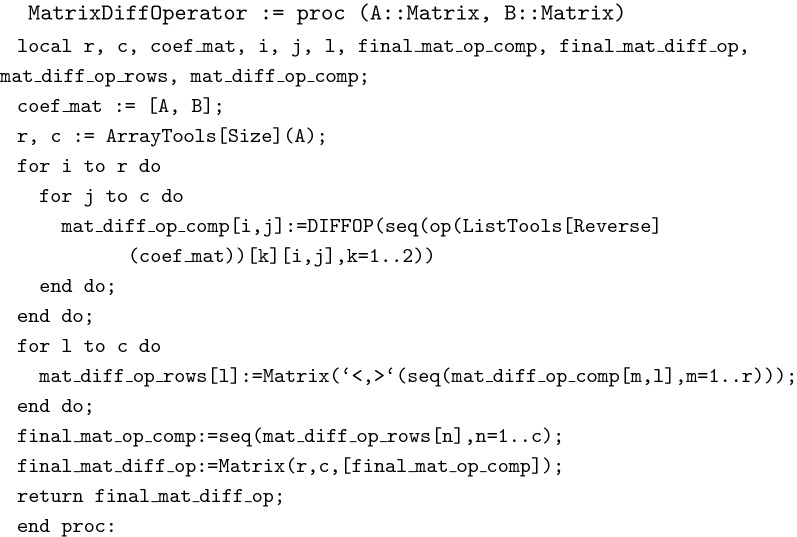


The function CanonicalMatrixSystem(A,B,f) produces the canonical form of a given DAEs, where *f* is a given vector forcing function.
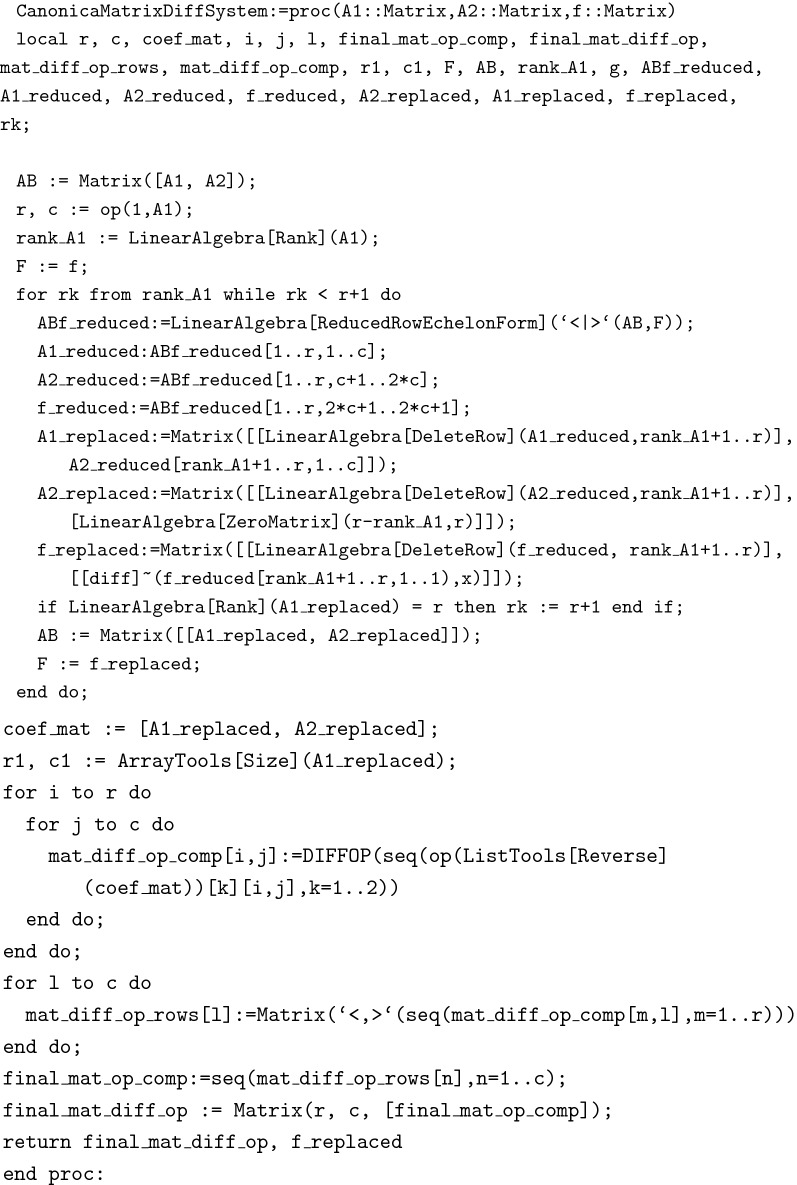


Finally ExactSolution(A,B,f) generates the exact solution of a given system.
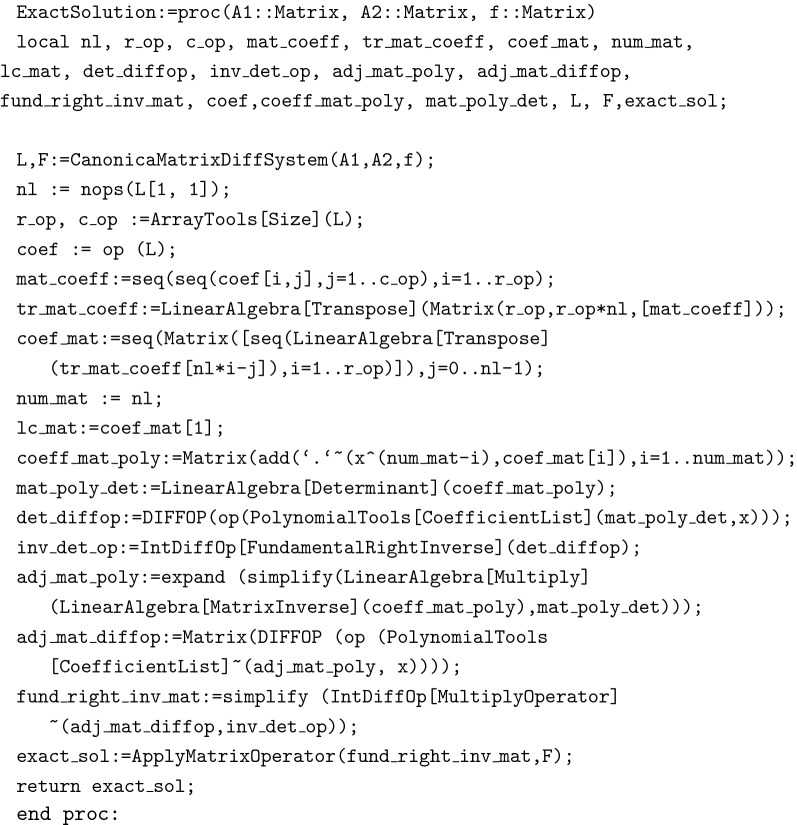


The following procedure, ApplyMatrixOperator(T,f), will help to verify the solution of the given problem.
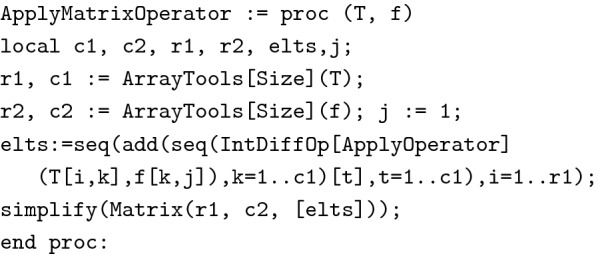


The implemented Maple package daeSolve is available with examples worksheet at http://www.srinivasaraothota.webs.com/research.

## Results

The following examples, shows the sample computations using the Maple package daeSolve as results of proposed maple package. We have also presented the comparison with existing method implemented in other mathematical software, namely Mathematica, in Example 5.

### *Example 5*

Consider an IVP with homogeneous initial conditions at zero.

8 $$\begin{aligned}y_1'-y_2'+2y_3'+y_1-2y_2+4y_3 &= x^2-x, \\y_2'-y_3'-y_2+y_3 &= e^{-x}, \\y_1-y_2+y_3 &= \sin x. \end{aligned}$$

Matrix representation of the given system in Maple is

- A := Matrix([[1,−1,2],[0,1,−1],[0,0,0]]);
- B := Matrix([[1,−2,4],[0,−1,1],[1,−1,1]]);
- f := Matrix([[x $^\wedge$ 2-x],[exp(-x)],[sin(x)]]);
- L := MatrixDiffOperator(A,B);

$$\begin{aligned} A:=\left[ \begin{array}{ccc} 1 &{} -1 &{} 2 \\ 0 &{} 1 &{} -1 \\ 0 &{} 0 &{} 0 \end{array} \right] \end{aligned}$$

$$\begin{aligned} B:=\left[ \begin{array}{ccc} 1 &{} -2 &{} 4\\ 0 &{} -1 &{} 1 \\ 1 &{} -1 &{} 1 \end{array} \right] \end{aligned}$$

$$\begin{aligned} f:=\left[ \begin{array}{c} x^2-x \\ e^{-x} \\ \sin (x) \end{array} \right] \end{aligned}$$

$$\begin{aligned} L:=\left[ \begin{array}{ccc} 1+{\texttt{D}}&{} -2-{\texttt{D}}&{} 4+2{\texttt{D}}\\ 0 &{} -1+{\texttt{D}}&{} 1-{\texttt{D}}\\ 1 &{} -1 &{} 1 \end{array} \right] \end{aligned}$$

The following command is used to generate the canonical form of the given IVP.

- CanonicaMatrixDiffSystem(A,B,f);

$$\begin{aligned} \left[ \begin{array}{ccc} {\texttt{D}}~&{}~ -2 ~&{}~ 4+{\texttt{D}}\\ 0 &{} -1+{\texttt{D}}&{} 1-{\texttt{D}}\\ {\texttt{D}}&{} -{\texttt{D}}&{} {\texttt{D}}\end{array} \right] , \left[ \begin{array}{c} x^2-x-\sin (x)+e^{-x} \\ e^{-x} \\ \cos (x) \end{array} \right] \end{aligned}$$

Here the first matrix denotes $$\tilde{L}=\left[ \begin{array}{ccc} {\texttt{D}}~&{}~ -2 ~&{}~ 4+{\texttt{D}}\\ 0 &{} -1+{\texttt{D}}&{} 1-{\texttt{D}}\\ {\texttt{D}}&{} -{\texttt{D}}&{} {\texttt{D}}\end{array} \right]$$ and second denotes $$\tilde{f} = \left[ \begin{array}{c} x^2-x-\sin (x)+e^{-x} \\ e^{-x} \\ \cos (x) \end{array} \right]$$. Now the following command is used to compute the exact solution of the given IVP.

- y := ExactSolution(A,B,f);

$$\begin{aligned} y:= \left[ \begin{array}{c} \frac{1}{2}e^x - \frac{1}{2}e^{-x}+\sin (x) \\ \frac{1}{30}e^{-2x}+\frac{1}{2}x^2-x+\frac{1}{2}-\frac{1}{5}\cos (x)-\frac{3}{5}\sin (x)-e^{-x}+\frac{2}{3}e^x \\ \frac{1}{30}e^{-2x} + \frac{1}{2}x^2-x+ \frac{1}{2} - \frac{1}{5}\cos (x)- \frac{3}{5}\sin (x)- \frac{1}{2}e^{-x} + \frac{1}{6}e^x \end{array} \right] \end{aligned}$$

One can also cross check the solution by substituting *y* into the given IVP as follows.

- ApplyMatrixOperator(L,y);

$$\begin{aligned} \left[ \begin{array}{c} x^2-x \\ e^{-x} \\ \sin (x) \end{array} \right] \end{aligned}$$

Using Mathematica (online computations from *wolframalpha.com*), we have a general solution with free parameters $c_1, c_2$ as follows

$$\begin{aligned} y_{1} (x) &= c_{2} e^{x} - \frac{1}{2}e^{{ - x}} + \sin x, \\ y_{2} (x) &= \frac{{21}}{{16}}\left( {c_{2} e^{x} - \frac{1}{2}e^{{ - x}} } \right) + \frac{1}{{64}}\left( c_{1} e^{{ - 2x}} + \frac{4}{3}c_{2} e^{{ - 2x}} (e^{{3x}} - 1) \right.\\ &\quad \left.+ \frac{2}{{15}}e^{{ - 4x}} (5(48e^{{4x}} (x - 1)^{2} - 33e^{{3x}} ) + 192e^{{4x}} \sin x - 96e^{{4x}} \cos x) \right) - \sin x, \\ y_{3} (x) &= \frac{5}{{16}}\left( {c_{2} e^{x} - \frac{1}{2}e^{{ - x}} } \right) + \frac{1}{{64}}\left( c_{1} e^{{ - 2x}} + \frac{4}{3}c_{2} e^{{ - 2x}} (e^{{3x}} - 1) \right.\\ &\quad \left. + \frac{2}{{15}}e^{{ - 4x}} (5(48e^{{4x}} (x - 1)^{2} - 33e^{{3x}} ) + 192e^{{4x}} \sin x - 96e^{{4x}} \cos x) \right) - \sin x. \\ \end{aligned}$$

After simplification, we have

$$\begin{aligned} y_1(x)&= c_2 e^x -\frac{1}{2} e^{-x} + \sin x, \\ y_2(x)&= \frac{1}{64} c_1 e^{-2 x} - \frac{1}{48} c_2 e^{-2 x} + \frac{4}{3} c_2 e^x + \frac{1}{2}x^2 - x - e^{-x} - \frac{3}{5} \sin x - \frac{1}{5} \cos x + \frac{1}{2} \\ y_3(x)&= \frac{1}{64}c_1 e^{-2 x} - \frac{1}{48} c_2 e^{-2 x} + \frac{1}{3}c_2 e^x + \frac{1}{2}x^2 - x - \frac{1}{2}e^{-x} -\frac{3}{5} \sin x -\frac{1}{5} \cos x + \frac{1}{2}. \end{aligned}$$

We can find the values of $c_1$ and $c_2$ by substituting $x=0$ and $y=0$. Now the exact solution is

$$\begin{aligned} y_1(x)&= \frac{1}{2}e^x - \frac{1}{2}e^{-x}+\sin x, \\ y_2(x)&= \frac{1}{30}e^{-2x}+\frac{1}{2}x^2-x+\frac{1}{2}-\frac{1}{5}\cos x-\frac{3}{5}\sin (x)-e^{-x}+\frac{2}{3}e^x \\ y_3(x)&= \frac{1}{30}e^{-2x} + \frac{1}{2}x^2-x+ \frac{1}{2} - \frac{1}{5}\cos x- \frac{3}{5}\sin x- \frac{1}{2}e^{-x} + \frac{1}{6}e^x. \end{aligned}$$

One can check that the exact solution obtained by Maple implementation and the Mathematica implementation is same. Using the Maple package, we computed the solution directly without any free parameters.

### *Example 6*

Consider an IVP given in [1, Example 2.8] with homogeneous initial conditions at zero. Authors computed the solution manually using the algorithm proposed. Here, we compute the exact solution using the Maple implementation.

9 $$\begin{aligned} \begin{pmatrix} 1 &{} -1 &{} 2 \\ 0 &{} 1 &{} -1 \\ 0 &{} 0 &{} 0 \end{pmatrix} u' + \begin{pmatrix} 1 &{} -2 &{} 4 \\ 0 &{} -1 &{} 1 \\ 0 &{} 0 &{} 1 \end{pmatrix} u = \begin{pmatrix} e^x \\ \cos x \\ \sin x \end{pmatrix}. \end{aligned}$$

Matrix differential operator *L* and forcing function *f* of (9) are

- A := Matrix([[1,-1,2], [0,1,-1], [0,0,0]]);
- B := Matrix([[1,-2,4], [0,-1,1], [0,0,1]]);
- f := Matrix([[exp(x)], [cos(x)], [sin(x)]]);
- L := MatrixDiffOperator(A,B);

$$\begin{aligned} A:=\left[ \begin{array}{ccc} 1 &{} -1 &{} 2 \\ 0 &{} 1 &{} -1 \\ 0 &{} 0 &{} 0 \\ \end{array} \right] \end{aligned}$$

$$\begin{aligned} B:=\left[ \begin{array}{ccc} 1 &{} -2 &{} 4\\ 0 &{} -1 &{} 1 \\ 0 &{} 0 &{} 1 \\ \end{array} \right] \end{aligned}$$

$$\begin{aligned} f:=\left[ \begin{array}{c} e^{x} \\ \cos (x) \\ \sin (x) \\ \end{array} \right] \end{aligned}$$

$$\begin{aligned} L:=\left[ \begin{array}{ccc} 1+{\texttt{D}}&{} -2-{\texttt{D}}&{} 4+2{\texttt{D}}\\ 0 &{} -1+{\texttt{D}}&{} 1-{\texttt{D}}\\ 0 &{} 0 &{} 1 \\ \end{array} \right] \end{aligned}$$

The canonical form of the given IVP is computed as,

- CanonicaMatrixDiffSystem(A,B,f);

$$\begin{aligned} \left[ \begin{array}{ccc} 1 + {\texttt{D}}~&{}~ -3 ~&{}~ {\texttt{D}}\\ 0 &{} -1+{\texttt{D}}&{} -{\texttt{D}}\\ 0 &{} 0 &{} {\texttt{D}}\end{array} \right] , \left[ \begin{array}{c} e^x-5\sin (x)+\cos (x) \\ \cos (x)-\sin (x) \\ \cos (x) \end{array} \right] \end{aligned}$$

Here $$\tilde{L}=\left[ \begin{array}{ccc} 1 + {\texttt{D}}~&{}~ -3 ~&{}~ {\texttt{D}}\\ 0 &{} -1+{\texttt{D}}&{} -{\texttt{D}}\\ 0 &{} 0 &{} {\texttt{D}}\end{array} \right]$$ and $$\tilde{f} = \left[ \begin{array}{c} e^x-5\sin (x)+\cos (x) \\ \cos (x)-\sin (x) \\ \cos (x) \end{array} \right]$$. Now the exact solution of the given IVP is

- y := ExactSolution(A,B,f);

$$\begin{aligned} y:= \left[ \begin{array}{c} \frac{5}{4}e^x - \frac{3}{4} e^{-x} - \frac{1}{2} \cos x - \sin x \\ \frac{1}{2}e^x - \frac{1}{2} \cos x + \frac{3}{2}\sin x \\ \sin x \end{array} \right] \end{aligned}$$

One can verify the solution by substituting *y* into the given IVP as follows.

- ApplyMatrixOperator(L,y);

$$\begin{aligned} \left[ \begin{array}{c} e^{x} \\ \cos (x) \\ \sin (x) \end{array} \right] \end{aligned}$$

## Limitations

The proposed algorithm presented in [1] for solving differential-algebraic equations is focused on the regular linear equations, hence the maple package presented in this paper is valid for the regular linear differential-algebraic equations. We have also presented an algorithm to check the regularity of the given problem.

## Abbreviations

IVP: initial value problem
DAE: differential-algebraic equation
